# Association of vertebral fractures with worsening degenerative changes of the spine: a longitudinal study

**DOI:** 10.1093/jbmr/zjae172

**Published:** 2024-10-17

**Authors:** Carrie Ye, William D Leslie, Mary L Bouxsein, Alyssa B Dufour, Ali Guermazi, Daniel Habtemariam, Mohamed Jarraya, Douglas P Kiel, Pradeep Suri, Elizabeth J Samelson

**Affiliations:** Department of Medicine, University of Alberta, Edmonton, AB T6G 2G3, Canada; Department of Epidemiology, Harvard T.H. Chan School of Public Health, Boston, MA 02115, United States; Department of Internal Medicine, University of Manitoba, Winnipeg, MB R3A 1R9, Canada; Department of Orthopedic Surgery, Harvard Medical School and Center for Advanced Orthopedic Studies, Beth Israel Deaconess Medical Center, Boston, MA 02215, United States; Musculoskeletal Research Center, Hinda and Arthur Marcus Institute for Aging Research, Boston, MA 02131, United States; Department of Medicine, Beth Israel Deaconess Medical Center and Harvard Medical School, Boston, MA 02215, United States; Department of Radiology, VA Boston Healthcare System, Boston University School of Medicine, Boston, MA 02118, United States; Musculoskeletal Research Center, Hinda and Arthur Marcus Institute for Aging Research, Boston, MA 02131, United States; Department of Radiology, Mercy Catholic Medical Center, Darby, PA 19023, United States; Musculoskeletal Research Center, Hinda and Arthur Marcus Institute for Aging Research, Boston, MA 02131, United States; Department of Medicine, Beth Israel Deaconess Medical Center and Harvard Medical School, Boston, MA 02215, United States; Rehabilitation Care Services, VA Puget Sound Health Care System, Seattle, WA 98108, United States; Seattle Epidemiologic Research and Information Center (ERIC), VA Puget Sound Health Care System, Seattle, WA 98108, United States; Department of Rehabilitation Medicine, University of Washington, Seattle, WA 98104, United States; Musculoskeletal Research Center, Hinda and Arthur Marcus Institute for Aging Research, Boston, MA 02131, United States; Department of Medicine, Beth Israel Deaconess Medical Center and Harvard Medical School, Boston, MA 02215, United States

**Keywords:** osteoporosis < diseases and disorders of/related to bone, osteoarthritis < diseases and disorders of/related to bone, general population studies < epidemiology, other < analysis/quantitation of bone, radiology

## Abstract

Vertebral compression fractures (VFs) and spinal degeneration are both common causes of back pain, particularly in older adults. Previous cross-sectional studies have shown a potential association between these entities, but there is limited evidence on the role of VFs in spinal degeneration. In this longitudinal study, we evaluated the association between prevalent VFs and the subsequent progression of facet joint osteoarthritis (FJOA) and intervertebral disc height narrowing (DHN), using data from the Framingham Heart Study Offspring and Third Generation Multi-Detector Computed Tomography study. Summary indices representing the total burden of each spinal parameter (VFs, DHN, and FJOA) were calculated for each individual. We hypothesized that prevalent VFs are associated with worsening spinal degeneration. Three hundred and seventy (31%) of 1197 participants had a baseline (prevalent) VF. The change in summary index of DHN over the follow-up period was significantly higher in those with vs without prevalent VF (difference in change in DHN 0.38, 95% CI 0.18 to 0.59, *p*<.001), but the change in summary index of FJOA was similar between those with and without prevalent VF. However, once adjusted for age, sex, cohort, smoking status, BMI, and baseline DHN, the change in summary index of DHN did not differ by prevalent VF status. There was a modestly higher change in the FJOA summary index in those with prevalent VFs compared to those without in the fully adjusted model (difference in change in FJOA 0.62, 95% CI −0.01 to 1.24, *p* = .054), driven primarily by those with severe (grade 3) VF (difference in change in FJOA 4.48, 95% CI 1.99-6.97). Moreover, there was greater change in the summary index of FJOA with increasing severity of prevalent VF (linear trend *p* = .005). Beyond the established morbidity and mortality associated with VFs, our study suggests that VFs may also lead to worsening spine osteoarthritis.

## Introduction

In 2020, low back pain affected 619 million people globally and was the leading cause of years lived with disability.^1^ Spinal degeneration and vertebral fractures (VFs) are common causes of low back pain. VFs are one of the most common skeletal fractures and reduce quality of life and survival in older adults.^2^ Several clinical characteristics have been associated with prevalent radiographic VFs, including older age, lower BMD, height loss, glucocorticoid use, greater weight, prior fracture, smoking, and back pain.^3^ The back pain resulting from VFs can lead to limited activities for a longer period of time than hip fractures.^4^

Facet joint osteoarthritis (FJOA) and disc height narrowing (DHN) are common degenerative changes seen in the spine, which increase with age and may play a particularly important role in older adults with back pain.^5^^,^^6^^,^^7^ There is considerable epidemiological and pathophysiological overlap between FJOA and DHN, with emerging evidence of the interdependence between DHN and FJOA.^8^ Less understood is the relationship between spinal degeneration and VF. Cross-sectional studies have shown conflicting results. Studies have demonstrated an inverse relationship between osteoporosis and osteoarthritis of the spine, leading to a common belief that osteoarthritis is “protective” against osteoporosis.^9–11^ However, other studies have found a positive association between osteoporotic fractures and osteoarthritis.^12–14^

While cross-sectional studies, done on clinical populations referred for spinal symptoms, have demonstrated a possible link between VFs and spinal degeneration, there is a gap in the literature examining the impact of prevalent VF on subsequent progression of spinal degeneration in a community setting, which can only be done through community-based longitudinal studies. VFs change the architecture of the spine,^15^ the metabolic microenvironment, and the direction and magnitude of forces through each level of the spine.^16^ We hypothesize that these changes may lead to progression of spinal degeneration (both DHN and FJOA). In this longitudinal study, we evaluated the association between prevalent VFs and changes in spinal degeneration over time using data from the Framingham Heart Study Offspring and Third Generation Multi-Detector Computed Tomography (CT) study.^17^

## Materials and methods

### Study design and participants

We used data collected from the longitudinal Framingham Heart Study Offspring and Third Generation Multi-Detector CT Study (Framingham MDCT Study).^18^ Members of the Offspring and Third Generation Cohorts included second-generation (plus spouses) and third-generation offspring of the original cohort of the Framingham Heart Study established in 1948.^19^ CT scans of the chest and abdomen were completed to assess coronary and aortic calcium at baseline in 2002–2005 and repeated at follow-up in 2008-2011, with an average interval of 6 yr.^17^^,^^18^ Exclusion criteria for the Framingham MDCT Study included pregnancy or baseline age less than 40 yr for females, age <35 yr for men, and weight >320 pounds (145 kg). We included all members of the Framingham MDCT Study who were at least 50 yr of age at the time of the baseline scans and who had both baseline and follow-up CT scans evaluable for radiographic assessment. Participants provided informed consent, and the Advarra Institutional Review Board for Human Research at Hebrew SeniorLife approved this study.

### CT imaging acquisition

CT imaging acquisition has previously been described in detail.^20^^,^^21^ Briefly, an eight-section multidetector CT unit (Lightspeed Ultra/Plus, General Electric Medical Systems, Milwaukee, WI, USA) was used for the baseline examination (2002-2005) and a 64-section multidetector CT unit (Discovery VCT, General Electric Medical Systems) was used for the follow-up examination (2008-2011). The availability of 64-slice CT in the follow-up visit allowed an improvement in the speed of acquisition, a decrease in radiation dose, and better spatial resolution. However, these differences with the baseline CT examination (acquired using an 8-slice scanner) were unlikely to affect the radiological comparability of the two time points with regard to assessment of VF and FJOA. Given the MDCT study was originally designed to assess the coronary arteries and the abdominal aorta, the CTs included a thoracic series (slice thickness = 2.5 mm) from the carina of the trachea to the diaphragm and an abdominal series where the L5/S2 junction was identified, and 60 contiguous CT slices (150 mm) were acquired above this point. Since the contrast and spatial resolutions of a CT examination are mainly dependent on the CT technology rather than the region of interest, CT evaluation of the spine can be easily performed from an abdominal CT.^22^

### Semi-quantitative scoring of VF, DHN, and FJOA

A single, trained musculoskeletal radiologist (MJ) used standardized protocols to evaluate VF, DHN and FJOA. The baseline and follow-up images were evaluated side-by-side with knowledge of chronology but blinded to clinical information. Imaging scoring in chronological order increases the sensitivity of detecting clinically relevant longitudinal changes.^23^ All three parameters were evaluated at each intervertebral level from T4/T5 to L4/L5 on baseline and follow-up scans. The reader assigned a semiquantitative (SQ) score for each trait: Grade 0 = none, Grade 1 = mild, Grade 2 = moderate, and Grade 3 = severe. To evaluate reliability, the reader assessed each parameter on two separate occasions for 30 individuals. Intraclass correlation coefficients for intra-reader reliability were 0.76-1.00 for VF, 0.80-1.00 for DHN, and 0.73-1.00 for FJOA.^20^^,^^24^

### Prevalent VF assessment

Prevalent VF at baseline CT evaluation was assessed by the same radiologist and graded using the Genant semi-quantitative method.^25^ No VF (Grade 0) was defined as <20% reduction in vertebral height, mild VF (Grade 1) as 20%-25% reduction in vertebral height, moderate VF (Grade 2) as 25%-40% reduction, and severe VF (Grade 3) as more than 40% reduction. Vertebral levels, which included Schmorl’s nodes, were considered to have VFs if there was associated global vertebral height loss. Vertebral remodeling related to degenerative disease and Scheuermann’s disease were not considered to be VFs. Participants were considered to have had a prevalent VF if they had at least one Grade 1 VF on their baseline CT. To account for both the number and grade of VFs in an individual, we calculated a baseline spinal deformity index (SDI) by summing the grades of all VFs across 13 spinal levels, yielding a range from 0 to 39.

### DHN assessment

DHN was assessed at baseline and follow-up using Videman’s approach.^26^ Each disc was compared to the disc immediately superior and defined as no DHN (Grade 0) if the disc height was greater than that of the disc immediately superior, mild DHN (Grade 1) if it was equal, moderate (Grade 2) if it was less, and severe (Grade 3) if the vertebral endplates were almost in contact. We calculated a baseline and follow-up summary index for DHN by summing the SQ scores across 13 spinal levels, yielding a range from 0 to 39.

### FJOA assessment

FJOA was graded bilaterally using the Framingham scale,^20^^,^^24^^,^^27^^,^^28^ which was developed based on criteria originally described by Pathria *et al.*^29^ It considers the severity of the following factors at each facet joint: joint space, osteophytosis, articular process hypertrophy, sclerosis, subarticular erosion, subchondral cystic change, and the presence of vacuum phenomenon. No FJOA (Grade 0) was defined as joint space ≥2 mm; mild FJOA (Grade 1) as joint space <2 mm and/or mild osteophytosis or hypertrophy of the articular process; moderate FJOA (Grade 2) as joint space <1 mm and/or moderate osteophytosis or hypertrophy of the articular process and/or mild subarticular bone erosions; and severe FJOA (Grade 3) as no joint space and/or severe osteophytosis, hypertrophy of the articular process, subarticular bone erosions, subchondral cysts, or vacuum phenomenon in the joints. We calculated a baseline and follow-up summary index for FJOA, summing the SQ scores across 26 spinal levels (13 for the right side and 13 for the left side), ranging from 0 to 78.

### Evaluation of longitudinal change in DHN and FJOA

Worsening (yes/no) of DHN and FJOA at each vertebral level was defined by an increase of at least one unit in the SQ scores for DHN and FJOA between baseline and follow-up at that level. This included new (at least one level increasing from Grade 0 to Grade 1 or greater during follow-up) or worsening DHN and FJOA.

For each individual, total changes in DHN and FJOA were calculated by subtracting the baseline summary index from the follow-up summary index.

### Covariates

Clinical parameters were assessed by clinical examination and standardized questionnaires administered at study visits closest to the time of the baseline CT acquisition. Weight was measured to the nearest 0.5 pounds using a balance beam scale, and height was measured to the nearest 0.25 inch using a stadiometer. Participants were categorized as current smokers if they reported smoking at least one cigarette per day within the past year, past smokers if they reported smoking cigarettes previously, but not within the past year, and never smokers if they have never smoked. Alcohol consumption was calculated as ounces per week by multiplying the average amount of alcohol in a single drink of beer, wine, or spirit by the average number of self-reported drinks per week.

Physical activity was assessed using the physical activity index (PAI) based on self-reported average number of hours per day performing various levels of physical activity.^30^^,^^31^ Medications and menopausal status were self-reported. Diabetes was defined as fasting plasma glucose >125 mg/dL (7.0 mmol/dL) or on treatment with insulin or oral hypoglycemic agents. BMI was calculated by dividing weight (kg) by height squared (m^2^).

### Statistical analysis

Summary statistics were used to describe baseline characteristics, stratified by prevalent VF status. Continuous variables were compared between those with and without prevalent VF using Student’s *t*-tests, and categorical variables were compared using Chi-square tests. DHN and FJOA summary indices were summarized as mean and SD at baseline and follow-up for the full cohort and stratified by prevalent VF status. Changes in DHN-summary index and FJOA-summary index were summarized as mean and SD for the full cohort and stratified by prevalent VF status. The distribution of prevalent VF, worsening DHN, and worsening FJOA was shown by vertebral level.

To examine the association between VF and spinal degeneration, we compared the difference in change in summary index in those individuals with and without prevalent VF. We used linear regression to assess the association between prevalent VF and change in DHN summary index and FJOA summary index using unadjusted models, age- and sex-adjusted models, and multivariable models additionally adjusted for cohort, smoking status, BMI, and baseline DHN summary index or FJOA summary index. These factors were included in the main model as they were considered to be potential confounders (associated with both the exposure and outcome).^32–34^ Due to missing information on physical activity for 5% of participants, we performed sensitivity analysis by including PAI in the main model. We assessed effect modification by sex and age with interaction terms. Stratified analyses were carried out if there was evidence of effect modification (*p*<.05). We evaluated the association between VF severity (defined as the highest grade of prevalent VF at any vertebral level) and change in DHN summary index and FJOA summary index by performing pairwise comparisons between mild (Grade 1), moderate (Grade 2), and severe (Grade 3) against no prevalent VF (Grade 0), along with assessment of linear trend. We also modeled the linear trend of baseline SDI and change in DHN summary index and FJOA summary index.

## Results

**Figure 1:**
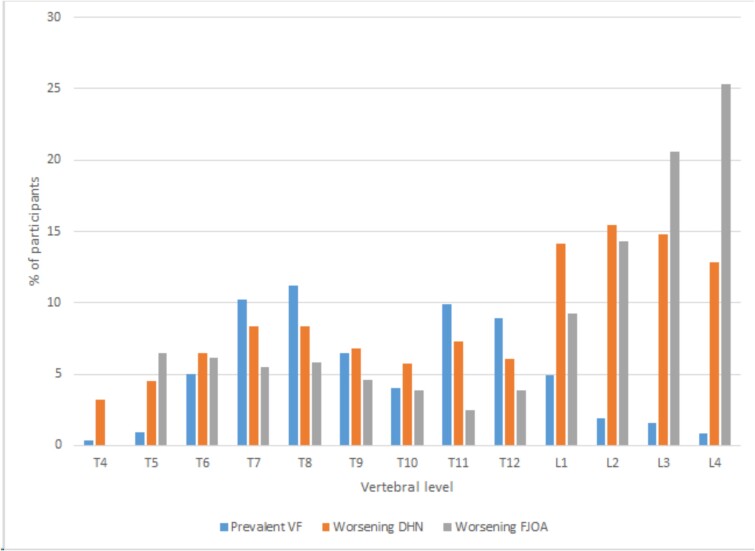
Prevalent VF and worsening DHN and FJOA over 6 years, by vertebral level. Worsening DHN and FJOA was defined as an increase of at least 1 unit of SQ score at the vertebral level from baseline to follow-up. Abbreviations: VF = vertebral fracture; DHN = disc height narrowing; FJOA = facet joint osteoarthritis.

The cohort included 1197 participants, 889 from the Offspring Cohort and 308 from the Third Generation Cohort. Three hundred and seventy individuals (31%) had a prevalent VF at baseline (293 with highest grade 1 at any of the spinal levels, 62 with grade 2, and 15 with grade 3). Those with prevalent VF were more likely to be male, older, have a higher BMI, be past or current smokers, drink more alcohol, and be more physically active (Table 1). Individuals with prevalent VF were also less likely to be on osteoporosis medication and more likely to have diabetes. Of the females in the cohort, those with prevalent VF were more likely to be postmenopausal. The percentages of participants with VF and worsening DHN and FJOA by vertebral level are presented in Figure 1. Worsening DHN and FJOA by vertebral level are further illustrated separately for those with and without prevalent VF in Supplementary Figure S1A and B. There was a bimodal distribution in VF with a peak in the mid-thoracic region and again in the lower thoracic region. Worsening of both DHN and FJOA during follow-up showed a small peak at the mid-thoracic region and a larger peak in the lumbar region. From the upper to the lower lumbar spine, FJOA increased, while DHN did not.

**Table 1:** Baseline characteristics by prevalent VF status.

Baseline characteristic	Total *n* = 1197	Prevalent vertebral fracture *n* = 370	No prevalent vertebral fracture *n* = 827	*p*-value
**Cohort** ** Offspring, *n*(%)** ** Third generation, *n*(%)**	889 (74) 308 (26)	330 (89) 40 (11)	559 (68) 268 (32)	<.001
**Female**	657 (55)	166 (45)	491 (59)	<.001
**Age y**	60 (9)	63 (9)	60 (8)	<.001
**BMI kg/m^2^**	28 (5)	29 (5)	28 (5)	<.001
**Smoking status** ** Never, *n*(%)** ** Past, *n*(%)** ** Current, *n*(%)**	490 (41) 604 (51) 101 (8)	133 (36) 206 (56) 30 (8)	357 (43) 398 (48) 71 (9)	.045
**Alcohol (oz/wk)**	2 (3)	3 (4)	2 (3)	<.001
**Physical activity index, *n*(%)**	37 (6)	38 (6)	37 (7)	<.001
**Osteoporosis medication, *n*(%)**	99 (8)	17 (5)	82 (10)	.002
**Post-menopausal (percent of females), *n*(%)**	483 (74)	145 (87)	338 (69)	<.001
**Diabetes, *n*(%)**	84 (7)	32 (9)	52 (6)	<.001

Abbreviation: BMI, body mass index.

^*^All values expressed as *n*(%) or mean (SD).

Missing: 3 BMI, 2 smoking status, 11 alcohol, 60 physical activity index, 3 menopause status (percent of females only), and 13 diabetes status.

The average DHN summary index was significantly higher in those with vs without VF at baseline (9.96 vs 6.29, *p*<.001) and at follow-up (11.42 vs 7.39, *p*<.001, Table 2). Similarly, the average FJOA summary index was significantly higher in those with vs without VF, at baseline (26.95 vs 22.31, *p*<.001) and at follow-up (29.91 vs 24.91, *p*<.001). The change in summary index of DHN over the follow-up period was significantly greater in those with prevalent VF than in those without prevalent VF (1.49 vs 1.10, *p*<.001), but the change in summary index of FJOA was similar between those with and without prevalent VF (3.01 vs 2.66, *p* = .257).

**Table 2:** Baseline, 6-yr follow-up, and change in summary indices for DHN and facet joint osteoarthritis, by prevalent VF status. Unadjusted means (SD) are shown.

**Timepoint**	**Prevalent vertebral fracture** ***N* = 370**	**No prevalent vertebral fracture** ***N* = 827**	***p*-value**
**Disc height narrowing summary index, mean (SD)** ^*^			
** Baseline**	9.96 (5.42) *n* = 369	6.29 (4.95) *n* = 826	**<.001**
** Follow-up**	11.42 (6.08) *n* = 370	7.39 (5.54) *n* = 826	**<.001**
** Change**	1.49 (1.90) *n* = 369	1.10 (1.58) *n* = 826	**<.001**
**Facet joint osteoarthritis summary index, mean (SD)** ^*^			
** Baseline**	26.95 (12.46) n = 369	22.31 (12.07) *n* = 821	**<.001**
** Follow-up**	29.91 (12.61) *n* = 370	24.91 (12.10) *n* = 826	**<.001**
** Change**	3.01 (5.30) *n* = 369	2.66 (4.85) *n* = 821	.257

^*^Baseline and follow-up summary indices were calculated for each person by summing the SQ scores (range, 0-3) across all spinal levels (13 for disc height narrowing and 26 for facet joint osteoarthritis). Change over 6 yr was calculated for each person by subtracting the baseline summary index from the follow-up summary index.

Table 3 presents associations between VF and changes in DHN and FJOA summary indices in both unadjusted and two models of adjustment. The change in summary index of DHN over the follow-up period was significantly higher in those with VF (0.38, *p*<.001), but the change in summary index of FJOA did not significantly differ between those with and without VF (0.35, *p* = .257). When adjusted for age and sex, the change in summary index of both DHN (0.27, *p* = .011) and FJOA (0.63, *p* = .049) was higher in those with VF, as compared to those without VF. After adjusting for age, sex, cohort, smoking status, BMI, and baseline DHN, the change in summary index of DHN did not differ between those with vs without VF (0.00, *p* = .988). However, there was a modestly greater change in the summary index of FJOA in those with VFs (0.62, *p* = .054), which very nearly met the significance cut-off of *p*<.05). Effect modification was not observed for sex and age in the fully adjusted models for DHN and FJOA (all interaction terms *p*>.05).

**Table 3:** Association between prevalent VF and change in summary indices of disc height narrowing and facet joint osteoarthritis over 6 yr.

	**Difference in change in summary index between those with and without** **prevalent vertebral fracture**	**95% CI**	***p*-value**
**Unadjusted**			
** Disc height narrowing**	0.38	0.18 to 0.59	**<.001**
** Facet joint osteoarthritis**	0.35	−0.26 to 0.97	.257
**Adjusted for age and sex**			
** Disc height narrowing**	0.27	0.06 to 0.48	**.011**
** Facet joint osteoarthritis**	0.63	0.00 to 1.26	**.049**
**Additionally adjusted for cohort, smoking status, BMI, and baseline summary index**			
** Disc height narrowing**	0.00	−0.21 to 0.22	.988
** Facet joint osteoarthritis**	0.62	−0.01 to 1.24	.054

BMI = body mass index. Bold = significant at a cutoff of *p*<0.05.

We found no significant difference in the change in DHN summary index by VF severity, after adjusting for sex, age, cohort, smoking status, BMI, and baseline DHN summary index (linear trend *p* = .700, Table 4). However, there was a greater change in FJOA with increasing severity of prevalent VF (linear trend *p* = .005), and on stratification by VF severity, there was only a significant association between severe VFs (Grade 3) and FJOA with an adjusted difference in change in FJOA summary index of 4.62 (95% CI 2.02-7.21). There was no significant association between baseline SDI and change in DHN (per unit increase in baseline SDI, adjusted difference in change in DHN SI 0.00, 95% CI −0.07 to 0.07, *p* = .994), but there was a 0.22 greater increase in FJOA SI for every unit increase in baseline SDI (95% CI 0.03-0.41, *p* = .024).

**Table 4:** Association between severity of prevalent VF and change in summary indices of disc height narrowing and facet joint osteoarthritis over 6 yr.

**Severity of prevalent vertebral fracture**	***n* = 1197** ***n* (%)**	**Adjusted** ^*^ **difference in change in summary index between individuals with and without prevalent vertebral fracture**	**95% CI**
**Disc height narrowing**			
** Grade 0**	827 (69.1)	Reference	Reference
** Grade 1**	293 (24.5)	0.01	−0.23 to 0.24
** Grade 2**	62 (5.28)	0.11	−0.32 to 0.53
** Grade 3**	15 (1.3)	−0.65	−1.49 to 0.18
** Linear trend *p*-value**		0.700	
**Facet joint osteoarthritis**			
** Grade 0**	827 (69.1)	Reference	Reference
** Grade 1**	293 (24.5)	0.43	−0.25 to 1.10
** Grade 2**	62 (5.2)	0.61	−0.65 to 1.87
** Grade 3**	15 (1.3)	4.48	1.99 to 6.97
** Linear trend *p*-value**		**.005**	

^*^Linear regression model including age, sex, cohort, smoking status, BMI, baseline summary index.

## Discussion

In this longitudinal Framingham Heart Study Offspring and Third Generation Multi-Detector CT study, we found that individuals with increasing severity of prevalent VF had more severe worsening of FJOA over 6 yr, with individuals with severe VF demonstrating significantly greater worsening of FJOA. In contrast, prior VF did not increase the risk of worsening of DHN.

VFs can alter the normal angles of the spine, including severe thoracic kyphosis or loss of lumbar lordosis.^35^ Changes in orientation of the facet joints and differences in the sagittal plane orientation of left vs right facet joints at the same spinal level (tropism), along with lumbosacropelvic morphology, such as sacral slope, sacral kyphosis, and lumbar lordosis, can change the amount and angle of loading through the facet joints, potentially leading to FJOA.^27^^,^^36^^,^^37^ Another theory known as the “spinal degenerative cascade” posits that changes related to degeneration in structures of the anterior spinal column—typically through intervertebral disc degeneration, such as DHN—can lead to dysfunction and/or greater forces applied through structures of the posterior spinal column, including the facet joints, which could manifest as FJOA. In this case, VFs may decrease the height of anterior spinal structures, leading to greater forces transferred through posterior spinal structures and consequent FJOA.^38^ While this study does not address the mechanism, a trend between increasing severity of prevalent VFs and increased worsening of FJOA was observed in our study.

A potential mechanism between VFs and the development or worsening of DHN is less clear. Researchers have hypothesized that since disc degeneration is associated with reduced nutrient supply and the blood supply from the vertebral body is the main source of disc nutrition, changes in vertebral body and end plate structures due to osteoporosis and/or VF may impact the nutrient supply and mechanical loading of the adjacent discs, thus leading to new or worsening disc degeneration.^16^^,^^39^ In our study, there was no association between prevalent VF and subsequent worsening of DHN after adjustment of potential baseline confounders, suggesting that the observed unadjusted association was largely reflecting the association of prevalent VF and worsening DHN with other baseline factors, such as age, sex, and baseline DHN.

Previous cross-sectional studies have examined the association between VF and degenerative spine changes, with conflicting results. In a cross-sectional study, investigators examined X-rays and MRIs of 98 men and women undergoing spinal surgery and found that VFs were associated with intervertebral disc degeneration.^14^ Conversely, a cross-sectional study based on X-rays of 410 postmenopausal women consulted for back pain found that disc space narrowing and osteophytes were inversely associated with VFs, after adjusting for age and weight.^10^ Not only are these findings contradictory, but also examining the association between two conditions, which are both more prevalent in aging women,^6^^,^^40^ and associated with back pain, can make confounding and selection bias difficult to avoid in cross-sectional studies. Furthermore, differences in definitions and methods for ascertaining aspects of spinal degeneration, including DHN and FJOA, make it challenging to compare results across studies.

A recent systematic review examining the link between vertebral osteoporosis and lumbar disc degeneration found that out of 19 clinical studies, 7 found a positive correlation between vertebral osteoporosis and lumbar disc degeneration, 8 found a negative correlation, and 4 did not find any correlation.^16^ Vertebral osteoporosis was assessed using a variety of BMD measurements in these studies, including DXA, quantitative CT, and even Hounsfield Unit values from CT instead of BMD. Lumbar disc degeneration was also defined and assessed differently across studies. The heterogeneity in ascertainment of these two parameters likely contributed to the contradictory results observed across these studies.

The relationship between prevalent spinal degeneration and subsequent development of VF has been previously studied. The longitudinal OFELY study found no association between osteophytes and subsequent development of VF, but did find a strong association between DHN and subsequent risk of developing VFs.^13^ A longitudinal study out of Japan found that the presence of spinal osteoarthritis (defined using the Kellgren–Lawrence grading method) was a risk factor for incident VFs.^41^ No studies have examined the effect of prevalent VFs on subsequent development or worsening of spinal degeneration.

One major strength of this study is the longitudinal nature of the Framingham Heart Study Offspring and Third Generation Multi-Detector CT study, which allows us to explore the impact of prevalent VFs on risk of new and worsening degenerative changes in the discs and facet joints. While we cannot attribute causation from this study, it provides insight into the potential effects of VFs on subsequent risk of developing or worsening spinal degeneration, compared to previous cross-sectional studies. Further, our community-based study cohort is not subject to the selection bias that is potentially present in clinical cohorts of patients with back pain requiring spine imaging. However, this longitudinal study is subject to survivor bias, which may have underestimated differences in spinal degenerative outcomes between those with and without VFs. We had a large sample size and information on important covariates, allowing adjustment for potential confounders. The number of individuals with severe VF was limited (*n* = 15, or 13%); however, we were still able to detect a linear trend between VF severity and worsening of FJOA.

Our study found that those with prevalent VFs had on average 0.62 greater increase in FJOA summary index over the average follow-up period of 6 yr compared to those without prevalent VF, driven largely by the small number of individuals with prevalent grade 3 VF, who had on average 4.48 greater increase in FJOA summary index. However, we do not know the clinical significance of these magnitudes of change. A previous cross-sectional study of 191 participants in the Framingham MDCT Study examined low back pain in relation to lumbar spine stenosis.^42^ However, information on low back pain symptoms was not available longitudinally nor for the full cohort in our study. This study is limited by a lack of information on back pain. As a result, it is difficult to fully understand the clinical significance of our findings and would be an important area for future research. Additionally, future studies examining the association between prevalent VF and the separate components of FJOA (joint space narrowing and osteophytes, which were not available in our dataset) could further our understanding of the underlying mechanism. Future in-depth vertebral-level evaluation of the observed person-level associations in this study will also enrich our understanding of the potential biomechanical factors at play between VF and spinal degeneration. Finally, the Framingham Heart Study Offspring and Third Generation cohorts are predominantly White individuals of European descent, limiting the generalizability of our results to other race and ethnic groups, which may have differing associations or magnitudes of effect size.^43^

Individuals who have suffered VFs experience more back pain even 12 yr after the VF.^44^ While treatment is most often focused on pain control in the acute period and prevention of subsequent VFs long term, perhaps more attention on detection, prevention, and management of spinal degeneration is warranted in these individuals.^45^ The results of this study contribute to our understanding of the long-term consequences and prognosis of VFs. Beyond the established morbidity and mortality associated with VFs, our study suggests that VFs, particularly severe VFs, may also lead to worsening spine osteoarthritis, adding still another reason to prevent osteoporotic VFs by screening for osteoporosis and treating when appropriate. If the findings of this study are confirmed in future studies, this important implication of VFs, particularly severe VFs, will be consequential for physicians to discuss with patients when counseling on the risks and benefits of treating osteoporosis.

## Supplementary Material

Supplementary_Materials_zjae172

## Data Availability

The procedure for requesting data from the Framingham Heart Study can be found at https://www.framinghamheartstudy.org/.
