# Supplementary material for: Association of vertebral fractures with worsening degenerative changes of the spine: a longitudinal study
Source: J Bone Miner Res. 2024 Oct 17;39(12):1744–51. doi: 10.1093/jbmr/zjae172 (PMC11638720; doi:10.1093/jbmr/zjae172)
Supplement: Supplementary_Materials_zjae172 [file supplementary_materials_zjae172.docx]

**eFigure 1. Worsening DHN and FJOA over 6 years, by vertebral level, in individuals with (A) and without (B) prevalent VF**

**A. Individuals with prevalent VF, N=370**


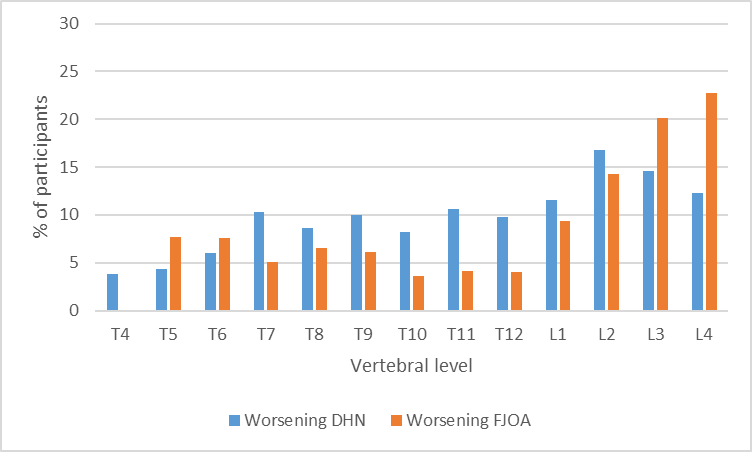


**B. Individuals without prevalent VF, N=827**

**
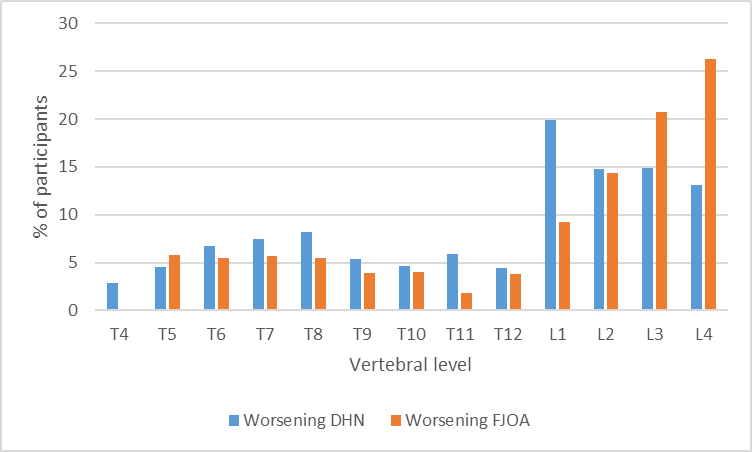
**

*Worsening DHN and FJOA was defined as an increase of at least 1 unit of SQ score at the vertebral level from baseline to follow-up. VF=vertebral fracture; DHN=disc height narrowing; FJOA=facet joint osteoarthritis.*
